# Assessment of competencies of clinical research professionals and proposals to improve clinical research in Portugal

**DOI:** 10.3389/fphar.2025.1578955

**Published:** 2025-04-08

**Authors:** Mónica Bogas, Joana Antas, Cátia Magalhães, Mafalda Revige, Liliana Guerra, Cheila Ribeiro, Rita Cunha Eça, Filipa Nunes, Ana Lopes, Luís Costa, Mónica Gonçalves, Jorge Pedrosa, Andreia Capela, Tiago Gregório, Patrícia Dias, Tiago Alfaro, Ana Pais, Rui Soares, Ana Queirós, Tiago Torres, Joana Assis, Joana Maia, Margarida Ferreira, Luís Horta, Rita Carreiro, João Almeida, Maria João Meireles, Carla Loução, Sofia António, Catarina Lopes, Pedro Coelho, Rita Costa, Margarida Santana, Nuno Sousa

**Affiliations:** ^1^ Roche Portugal, Amadora, Portugal; ^2^ AICIB – Agency for Clinical Research and Biomedical Innovation, Porto, Portugal; ^3^ Learning Health, Hospital da Luz Lisboa, Lisboa, Portugal; ^4^ Centro de Investigação Clínica, ULS Santa Maria, Lisboa, Portugal; ^5^ 2CA-Braga - Centro Clínico Académico de Braga, Braga, Portugal; ^6^ Unidade de Investigação e Ensaios Clínicos, ULS Gaia e Espinho EPE, Vila Nova de Gaia, Portugal; ^7^ Unidade de Inovação e Desenvolvimento, ULS Coimbra EPE, Coimbra, Portugal; ^8^ Instituto Português de Oncologia de Coimbra Francisco Gentil, Coimbra, Portugal; ^9^ Unidade de Ensaios Clínicos do Centro Académico Clínico, ICBAS/ULS Santo António, Porto, Portugal; ^10^ Centro de Investigação do Instituto Português de Oncologia do Porto (CI-IPOP), Porto, Portugal; ^11^ Centro de Investigação, ULS São José EPE, Lisboa, Portugal

**Keywords:** clinical research, core competency, professional development, self-assessment, the Joint Task Force for Clinical Trial Competency, clinical trials

## Abstract

**Background:**

Clinical studies are coordinated by multidisciplinary teams, which often lack adequate training and competencies. In this study, ROCHE and AICIB (Agency for Clinical Research and Biomedical Innovation) conducted a self-assessment survey aiming to evaluate the competency of clinical research professionals to conduct clinical research in Portugal and promote the identification of key actions to address priority gaps.

**Methods:**

Clinical research professionals from 10 Portuguese centres answered an electronic survey, adapted and translated from the Joint Task Force for Clinical Trial Competency (JTFCTC) framework. Representatives of the centres, ROCHE and AICIB held a meeting to discuss the survey results, identify priority gaps and propose recommendations.

**Results:**

A total of 109 participants answered the questionnaire with the following national geographical distribution: North (*n* = 46), Centre Region (*n* = 16), and Lisbon metropolitan area (*n* = 47). A considerable proportion were Investigators (44.0%) and had more than 10 years of experience (34.9%). The eight JTFCTC Domains scored under 60% in the level of knowledge, with Investigators achieving overall higher scores. To address these gaps, key actions were proposed, such as enhancing training and educational opportunities, fostering collaboration and networking, and investing in infrastructure and resources.

**Conclusion:**

This study was the first to assess clinical trial competency in Portugal, registering a high participation rate. The study highlights the need to develop a national plan of action, in a collaborative effort, between clinical research centres, universities, industry, regulatory authorities, national agencies, and patient organizations. This will not only contribute to elevate the quality of studies but also improve compliance with international standards, ultimately benefiting both researchers and patients.

## 1 Introduction

Over the past few decades, the growth of randomized clinical trials has contributed to mainstreaming the concept of evidence-based medicine (Evidence-based medicine, 1992). These studies are highly regulated, requiring strict adherence to complex protocols and coordination by multidisciplinary teams. The proper design and conduct of clinical trials, in accordance with Good Clinical Practices (GCPs), is essential to maintain ethical standards and generate high-quality evidence. Furthermore, the growing complexity of study designs in an increasingly competitive and demanding field underscores the urgent need to invest in more qualified and well-prepared professionals. However, concerns have arisen regarding the minimal educational requirements, competencies, or adequate training needed to become a clinical research professional (Sonstein et al., 2014). This emphasized the need for comprehensive training programs for new professionals and continuous competency development to ensure the integrity and success of clinical trials.

In 2014, the Joint Task Force for Clinical Trial Competency (JTFCTC) developed a Core Competency Framework comprising eight domains of knowledge to define key skills and competencies necessary to conduct clinical research in compliance with ethical, safety and high-quality standards (Sonstein et al., 2014). After the Multi-Regional Clinical Trials Center of Brigham and Women’s Hospital and Harvard (MRCT Center) assumed responsibility for the Joint Task Force (JTF) in 2017, the original competency framework has gained broader recognition, and has since been updated, expanded to other contexts, and translated into multiple languages (Sonstein et al., 2016; Sonstein and Jones, 2018a; Sonstein et al., 2022a; Sonstein et al., 2022b; Sonstein et al., 2024). Several initiatives have applied and adapted the JTFCTC to various contexts, such as evaluation of competencies and accreditation, onboarding and training, human resources management, and for professional valorisation (Dobrova et al., 2022; Deeter et al., 2023; Sundquist et al., 2023; Yakubov et al., 2023; Cranfill et al., 2023; Besel et al., 2023; Snyder et al., 2024; Jones et al., 2024).

In Portugal, there is significant potential for growth in clinical research and in clinical studies. In 2023, the country had 551 health researchers (in full-time equivalent) per million inhabitants (5th in a total of 82 countries), contributing to 3.9% of total scientific publications produced by the European Union (11th in a total of 27 EU members) (WHO, 2023; DGEEC, 2023). Additionally, the most recent report from the European Federation of Pharmaceutical Industries and Associations (EFPIA) indicates that Portugal, along with Greece and Spain, contrasts with the trend of decreasing clinical trial starts observed in Northern and Western European countries (EFPIA EFoPIaA, 2024). However, there is limited data on the competencies of clinical research centres and their professionals. In line with trends observed in other countries (Sundquist et al., 2023; Knapke et al., 2022a; Knapke et al., 2022b), the management and quality of the clinical studies are impacted by the small number of dedicated and qualified professionals, high employee turnover of supporting staff, and reported gaps in competencies amongst investigators and site staff. Additionally, the professionals demonstrate concerns about precarious work conditions, limited opportunities for career progression, professional development, and access to adequate training programs (Borges-Carneiro et al., 2024; Zimbarra et al., 2022). The implementation of a competency-based job framework has been shown to decrease the turnover within clinical research professionals (Stroo et al., 2020).

In a collaborative work, Roche Portugal and AICIB (Agency for Clinical Research and Biomedical Innovation) aimed to conduct a self-assessment survey to evaluate the knowledge and competencies required for developing and conducting clinical studies in reference clinical research centres in Portugal. In addition, this initiative aimed to foster discussions among the participating centres to identify key actions for addressing the gaps identified as the uppermost priority.

This study provides a comprehensive overview of the knowledge and competencies required for clinical research within the Portuguese clinical context, along with actionable solutions to guide the development of a national action plan for implementation by clinical research centres. Moreover, it provides a Portuguese translated adaptation of the JTFCTC framework, serving as a valuable reference for other Portuguese clinical research centres and Portuguese speaking countries. Importantly, the proposed measures can also guide national and international centres with similar gaps.

## 2 Materials and methods

ROCHE and AICIB developed a Core Competency Framework derivative from the Leveled Core Competency Framework for the Clinical Research Professional Version 3.1 by the JTF and the MRCT Center licensed under CC BY-NC-SA 4.0. This adapted framework is a Portuguese translation approved by the MRCT Center (https://mrctcenter.org/clinical-trial-competency/framework/portuguese/) and assesses competencies in eight domains of knowledge (1. Scientific Concepts and Research Design; 2. Ethical and Participant Safety Considerations; 3. Investigational Products Development and Regulation; 4. Clinical Study Operations (Good Clinical Practice); 5. Study and Site Management; 6. Data Management and Informatics; 7. Leadership and Professionalism and 8. Communications and Teamwork) as described previously (Sonstein S. and Jones C., 2018), with minor adjustments to align with the EU context. In addition, a “no knowledge/not applicable to the role” level was added to the Basic, Skilled and Advanced levels. The scoring system for each question was defined as follows: 0 (no knowledge/not applicable to the role), 1 (Basic/Fundamental level), 2 (Intermediate/Skilled level) and 3 (Advanced/Expert level).

Ten clinical research centres in mainland Portugal were selected to participate in the survey based on their experience in clinical research, high volume of clinical studies, as well as established relationship with Roche Portugal. These centres have experience in conducting clinical trials, observational studies, real-world evidence (RWE) studies, and investigator-initiated trials (IITs) across various therapeutic areas, conducting most of the studies at the national level. Centres were contacted between April and May 2024, in-person meetings were held to clarify details, and an electronic survey was posteriorly sent by email. Each centre was responsible for internally distributing the survey, ensuring that participation was both optional and confidential. In addition to answering on their core competency knowledge, participants were asked to provide demographic information on their professional role (Investigator, Clinical Research Coordinator, Research Nurse, Research Pharmacist, or Other) and years of experience on the role (≤2, 3–5, 6–10 or >10 years), following similar grouping criteria used previously (Sonstein SA. et al., 2022). Participants who answered “other” were not asked to specify their role to ensure confidentiality of their answers, as this category can include roles performed by a single person of the research team. Data was collected and presented to representatives of the clinical research centres by Roche and AICIB during an in-person meeting held on June 6, 2024. Following the presentation of the results, participants were divided into four workgroups, each led by a moderator. Over approximately 90 min, each group discussed two specific domains, aiming to identify the highest-priority gaps and propose potential solutions, which were later discussed amongst all.

## 3 Results

### 3.1 Sample characterization

**FIGURE 1:**
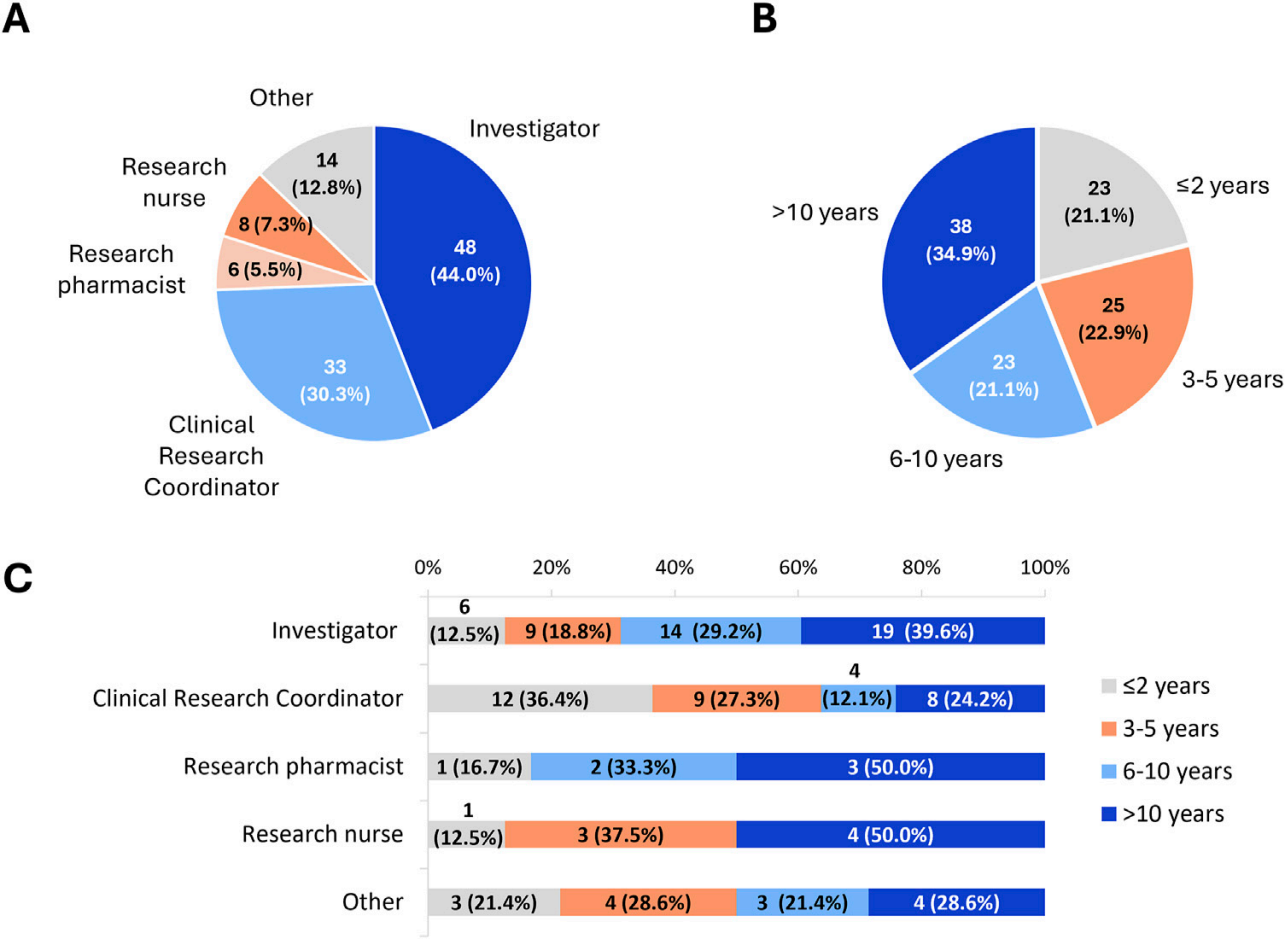
Characterization of the sample population completing the self-assessment questionnaire **(A)** by role and **(B)** by years of experience, and **(C)** distribution of each role according to years of experience. Data are shown as absolute and relative frequencies.

A total of 109 participants representing 10 Portuguese centres answered the questionnaire, being 5 clinical research centres located in the north (*n* = 46 participants), 2 in the centre region (*n* = 16 participants) and 3 in the Lisbon metropolitan area (*n* = 47 participants). Nearly all centres were part of the national state-funded healthcare (*n* = 9 [90%]). A considerable proportion of participants were Investigators (44.0%), including Principal Investigators and sub-Investigators. Other professionals were Clinical Research Coordinators (30.3%), Research Nurses (7.3%), Research Pharmacists (5.5%), or Other (12.8%) (Figure 1A). Regarding the years of experience, distribution was balanced, with around one-third having more than 10 years of experience (34.8%) (Figure 1B). A considerable proportion of Investigators (39.6%), Research Pharmacists (50.0%) and Research Nurses (50.0%) performed clinical investigation for longer than 10 years, whereas only 24.2% of Clinical Research Coordinators had that level of experience. Participants performing other roles in clinical research were evenly distributed in terms of their experience (Figure 1C).

### 3.2 Knowledge and competencies in all domains

Table 1 presents the average score and the maximum possible score in each domain. The level of knowledge was under 60% in all domains. The average score was higher for Scientific Concepts and Research Design (Domain 1, 56.5%), Communications and Teamwork skills (Domain 8, 56.1%), and Ethical and Participant Safety considerations (Domain 2, 55.8%). In contrast, self-assessed competencies revealed more gaps in Study and Site Management (Domain 5, 39.2%), Data Management and Informatics (Domain 6, 42.8%), and Investigational Products Development and Regulation (Domain 3, 43.3%).

**TABLE 1:** Average self-assessed competency score and maximum possible score by domain.

Domains	Average score	Maximum score
1. Scientific Concepts and Research Design	8.5 (56.5%)	15 (100%)
2. Ethical and Participant Safety Considerations	13.4 (55.8%)	24 (100%)
3. Investigational Products Development and Regulation	9.1 (43.3%)	21 (100%)
4. Clinical Study Operations (Good Clinical Practice)	14.4 (48.1%)	30 (100%)
5. Study and Site Management	8.2 (39.2%)	21 (100%)
6. Data Management and Informatics	5.1 (42.8%)	12 (100%)
7. Leadership and Professionalism	6.1 (50.8%)	12 (100%)
8. Communications and Teamwork	6.7 (56.1%)	12 (100%)

Data are shown as score (score in percentage).

### 3.3 Competency by professional role

Self-assessed competency was analysed by role in clinical research (Table 2). Overall, Investigators scored higher, particularly in the domains assessing Scientific Concepts and Research Design (Domain 1, 71.0%), Ethical and Participant Safety Considerations (Domain 2, 66.4%) and Communications and Teamwork (Domain 8, 63.4%). On the opposite, competencies in Study and Site Management (Domain 5) and Leadership and Professionalism (Domain 7) presented slightly higher scores for participants who had other roles in research.

**TABLE 2:** Average self-assessed competency scores by role.

	Investigator	Clinical research coordinator	Research pharmacist	Research nurse	Other
1. Scientific Concepts and Research Design	10.6 (71.0%)	6.4 (42.6%)	6.0 (40.0%)	7.6 (50.8%)	7.4 (49.5%)
2. Ethical and Participant Safety Considerations	15.9 (66.4%)	11.8 (49.1%)	9.0 (37.5%)	10.4 (43.2%)	12.1 (50.3%)
3. Investigational Products Development and Regulation	9.7 (46.2%)	8.6 (40.8%)	9.2 (43.7%)	7.8 (36.9%)	9.0 (42.9%)
4. Clinical Study Operations (Good Clinical Practice)	15.4 (51.2%)	14.2 (47.4%)	14.3 (47.8%)	12.1 (40.4%)	13.1 (43.6%)
5. Study and Site Management	9.0 (42.8%)	7.3 (34.9%)	7.7 (36.5%)	4.0 (19.1%)	10.4 (49.7%)
6. Data Management and Informatics	5.9 (49.5%)	5.0 (41.7%)	3.5 (29.1%)	3.5 (29.1%)	4.4 (36.3%)
7. Leadership and Professionalism	6.3 (52.3%)	6.2 (51.8%)	5.2 (43.0%)	4.5 (37.5%)	6.5 (54.2%)
8. Communications and Teamwork	7.6 (63.4%)	6.2 (51.3%)	6.7 (55.5%)	5.1 (42.7%)	6.1 (50.6%)

Data are shown as score (score in percentage).

### 3.4 Competency by experience

When considering the years of experience in clinical research, there was an overall trend for a rise in self-assessed knowledge and competencies as years of experience increased (Table 3).

**TABLE 3:** Average self-assessed competency scores by years of experience.

	≤2 years	3–5 years	6–10 years	>10 years
1. Scientific Concepts and Research Design	6.6 (43.8%)	8.7 (58.1%)	8.7 (58.3%)	9.3 (61.9%)
2. Ethical and Participant Safety Considerations	11.3 (47.3%)	13.6 (56.8%)	12.5 (52.0%)	15.0 (62.6%)
3. Investigational Products Development and Regulation	7.0 (33.1%)	9.4 (44.9%)	8.5 (40.6%)	10.5 (50.1%)
4. Clinical Study Operations (Good Clinical Practice)	11.4 (38.0%)	14.6 (48.8%)	13.7 (45.5%)	16.6 (55.3%)
5. Study and Site Management	5.6 (26.5%)	9.0 (42.7%)	7.5 (35.8%)	9.8 (46.6%)
6. Data Management and Informatics	4.7 (38.8%)	4.9 (40.7%)	4.7 (39.1%)	5.9 (48.9%)
7. Leadership and Professionalism	5.1 (42.7%)	6.0 (50.3%)	5.7 (47.8%)	6.9 (57.7%)
8. Communications and Teamwork	5.5 (45.6%)	6.3 (52.3%)	7.0 (58.3%)	7.6 (63.6%)

Data are shown as score (score in percentage).

### 3.5 Priority gaps and actions for improvement

The results of the survey were presented to representatives of the clinical research centres by Roche and AICIB. After, workgroups were formed to facilitate discussions focusing on assessing gaps, identifying the highest-priority issues, and proposing potential solutions for improvement.

#### 3.5.1 Assessment and gap analysis

Although the survey aimed to evaluate competencies in the development and conduct of clinical studies, it was adapted from the JTFCTC framework and thus, may not fully align with the context of clinical research in Portugal. Most research activities in Portugal are industry-driven, and thus many of the competencies assessed in the survey, namely, in Domains 2, 3, and 6, are the responsibility of the Sponsor or Contract Research Organizations (CROs). Considering this, workgroups identified and discussed the highest priority gaps in competencies that are the responsibility of clinical research centres in the context of clinical research in Portugal (Table 4).

**TABLE 4:** Priority competency gaps identified for each domain of knowledge.

	Priority competency gaps
1. Scientific Concepts and Research Design	⁃ Apply principles of biomedical science to investigational product discovery and development and health-related behavioural interventions
2. Ethical and Participant Safety Considerations^a^	⁃ Differentiate between standard of care and clinical study activities ⁃ Define the concepts of “clinical equipoise” and “therapeutic misconception” as they relate to the conduct of a clinical study ⁃ Apply relevant national and international principles of human subject protections and privacy throughout all stages of a clinical study
3. Investigational Products Development and Regulation^a^	⁃ Describe the pre- and post-approval safety reporting requirements of regulatory agencies
4. Clinical Study Operations (Good Clinical Practice)	⁃ Describe the role and purpose of clinical study audits ⁃ Describe the roles and responsibilities of the clinical investigation team as defined by GCP guidelines ⁃ Evaluate design, conduct and documentation of clinical studies as required for compliance with GCPs
5. Study and Site Management	⁃ Develop and manage the functional and operational efficiencies and personnel resources necessary to conduct a clinical study ⁃ Describe the management and training approaches to mitigate risk to improve clinical study conduct ⁃ Develop and implement strategies to manage participant recruitment, retention, compliance and track study activities
6. Data Management and Informatics^a^	⁃ Describe best practices and resources required for standardizing data collection, capture, management, analysis, and reporting ⁃ Describe, develop, and implement processes for data quality assurance
7. Leadership and Professionalism	⁃ Identify ethical and professional conflicts associated with the conduct of clinical studies and implement procedures for their prevention or management ⁃ Identify and apply the professional guidelines and codes of ethics that apply to the conduct of clinical research ⁃ Describe the impact of regional diversity and demonstrate cultural competency in clinical study design and conduct
8. Communications and Teamwork	⁃ Describe the importance of team science and methods necessary to work effectively with cross-functional, multidisciplinary and interprofessional research teams, which may include external partners ⁃ Effectively communicate the content and relevance of clinical research findings to colleagues, advocacy groups and the non-scientific community ⁃ Describe the components of a traditional scientific publication

^a^ Several competencies evaluated in this domain were considered the responsibility of the Sponsor or the CRO.

The discussion consolidated initiatives to guide the development of an action plan, categorized into three main areas: Training and Education, Infrastructure and Resources, and Collaboration and Networking, each described in detail below.

#### 3.5.2 Training and education

Promotion of opportunities for training and accreditation of professionals was considered of utmost priority by all workgroups. These processes should ideally take place during the onboarding of new professionals, allowing them to select the appropriate level of training.

To address the gaps in Domain 1, a training system should be encouraged by providing incentives, such as increased funding, for those with certification.

To address the gaps in Domain 2, it was proposed to strengthen the inclusion of research pharmacists, who were particularly less confident in the identified core competencies. Training and accreditation of professionals was also considered important. In addition, measures to increase patient literacy and wider dissemination within social and media networks should be implemented to guarantee that patients, particularly the most vulnerable, are better informed.

To address the priority gap in reporting safety information to regulatory agencies before and after market introduction (Domain 3), specialized training, dedicated professionals, and a continuous evaluation should be required.

To overcome gaps identified in Domain 4, namely, the inadequacy in describing team roles and responsibilities, it was suggested to implement practical, personalized training and have professionals from distinct categories describe their functions to the team. Of note, the same professional often performs several activities in the centre.

To address the gaps in Domain 5, it was suggested to promote development and multidisciplinary training at national level to enhance competencies in managing functional and operational efficiencies, as well as human resources.

Identifying professional conflicts and understanding professional ethical codes were considered priority competencies in Domain 7. E-learning training to increase adherence, formal personalized onboarding programs, regardless of role and responsibilities, and mentoring programs are potential measures to develop this knowledge. Reference centres and speakers should use practical cases illustrating problems and challenges that arise in the issue of professionalism during training.

Lastly, in Domain 8, developing competencies to work effectively in cross-functional, multidisciplinary teams was considered a priority, for which shadowing colleagues in different roles and responsibilities can be useful. Moreover, to address gaps in communication skills, fomenting opportunities to present work to patient associations and the general public, offering workshops to improve manuscript writing, and recognizing high-quality communications can enhance clinical research professionals’ ability to disseminate their findings.

#### 3.5.3 Infrastructure and resources

Another key area of development focuses on the reorganization of infrastructure and resources. Thus, involvement at the high institutional level and top management is crucial to execute measures that incentivize research, including directives, hospital activity plans, and protected time for research.

To improve reporting of safety information to regulatory agencies pre- and post-approval (Domain 3), each patient should be identified in the hospital system with a universal or national code throughout the trial. Moreover, strategies to improve the internal notification circuit in the hospital, such as an SOS button, can be implemented. Importantly, patients should be trained to adequately notify safety information during clinical studies.

Key priority gaps in Domain 4 included the inability to adequately describe the role and purpose of clinical study audits and to evaluate the study design, conduct, and documentation of clinical studies, as required for compliance with GCPs. Centres demonstrating the poorest performance in internal or external audits should receive focused attention. In this context, audit results should serve as a basis for the revision and improvement of standard operating procedures (SOPs).

To address gaps in developing and managing functional and operational efficiencies and personnel resources necessary to conduct clinical studies (Domain 5), centres need clear guidance on determining the appropriate staff size (*e.g.*, Clinical Research Coordinators) to support the typical volume of studies conducted. It was also considered a priority to enhance the competencies in risk mitigation and in developing strategies that improve study quality and participant recruitment and retention.

Knowledge gaps in adequate data management, accurate data entry, and query management were considered a priority to address (Domain 6). Institutional and individual program recognition of compliance can help incentivize best practices for standardized data collection, management, analysis, and reporting. These measures may include specialized training initiatives, internships, opportunities for conference participation, or financial support.

Lastly, it was considered important to improve cultural competency in the design and conduct of clinical studies (Domain 7). The creation of a checklist to identify potential issues and the collection of data on demographic regional alterations associated with migration can help eliminate bias and ensure cultural and regional appropriateness.

#### 3.5.4 Collaboration and networking

As part of professional development and multidisciplinary training, on-site events should be organized at the national level to facilitate knowledge sharing among professionals in diverse roles, while also fostering collaboration and networking. These training and professional development opportunities are especially important at the national level, given the limited availability of online resources in Portuguese. Furthermore, clinical research centres should coordinate to streamline their training programs, to minimize redundancy while enhancing opportunities for diverse professional development.

AICIB was established as a governmental initiative to advance clinical research and innovation. The national online platform that AICIB is currently developing will contribute to connect stakeholders, stimulate collaboration, centralize various services, and enhance Portugal’s international competitiveness in the field. Additionally, working groups with key national and international stakeholders can advance collaborative priority initiatives to strengthen national clinical research, including those promoted and supported by AICIB.

## 4 Discussion

This study was the first to assess clinical trial competency in Portugal. The 10 participating centres were chosen based on their extensive experience in conducting clinical trials, observational studies, RWE studies, and IITs. A total of 109 clinical research professionals answered the survey, an impressively high number relative to the country’s population, comparatively to studies performed globally or in other countries. For example, an electronic global survey registered only 661 completed surveys (Sonstein et al., 2022a) and similar studies conducted in Canada and Ukraine, countries with more clinical research units and higher populations, collected 40 and 186 completed questionnaires, respectively (Dobrova et al., 2022; Sundquist et al., 2023).

Overall, the areas of knowledge that scored higher were Scientific Concepts and Research Design, Communications and Teamwork, and Ethical and Participant Safety Considerations. In contrast, greater competency gaps in Study and Site Management, Data Management and Informatics, and Investigational Products Development and Regulation were observed. These results are in line with previous reports showing lower scores in the same domains (Sonstein et al., 2022a; Dobrova et al., 2022; Sundquist et al., 2023). The self-perceived higher competency in Scientific Concepts and Research Design is likely explained by the high proportion of Investigators. Indeed, 44.0% of the participants were Investigators, and a significant proportion of them (39.6%) had more than 10 years of experience in conducting clinical studies. It is not surprising that Investigators presented higher confidence in these competencies, as these are directly related to their functions, whereas gaps were identified in competencies that are not typically of their responsibility. Regarding the remaining roles, the overall lower scores, even in functions of their responsibility, are likely justified by the high employee turnover and less years of experience in clinical research. In this survey, 36.4% of Clinical Research Coordinators have been in their role for less than 2 years. These observations reflect the urgent need to implement measures for attracting, training, and retaining support staff (Knapke et al., 2022a; Knapke et al., 2022b). Globally, it is recognised that several factors contribute to high staff turnover, including irregular work schedules, excessive workloads, low organizational commitment, and dissatisfaction with compensation or career advancement opportunities. These challenges in staff retention aggravated during the COVID-19 pandemic, as working conditions for healthcare professionals deteriorated (Freel et al., 2023; Sun et al., 2023). Moreover, rising quality standards, increased bureaucracy, and the complexity of study designs have led centres to respond to the demands in specialized human resources and to adapt their infrastructures (Stabile et al., 2023). In the latter study, Italian Clinical Research Coordinators pointed the lack of institutional recognition, professional role identity and progression opportunities, both at contractual and competence level, as reasons for excessive turnover (Stabile et al., 2023).

Lack of professional recognition, inadequate training, and no protected time for research are concerns expressed by clinical research professionals in Portugal (Borges-Carneiro et al., 2024; Zimbarra et al., 2022). In this study, centres’ representatives emphasized that certification and accreditation are key areas for improvement. In this context, it is critical to discuss if mandatory certification for clinical research professionals should be implemented to enhance the quality of work in clinical studies. In the aforementioned global electronic survey, professionals certified by recognized professional bodies, such as the Association of Clinical Research Professionals (ACRP) and Society of Clinical Research Associates (SOCRA) scored higher in all domains (Sonstein et al., 2022a). However, many clinical research certifications require significant financial investment and time commitment, which can be a burden for professionals and centres. To avoid discrepancies in rigor and quality, the implementation of standardized national training requirements should also be discussed. Centres, academic institutions, national agencies, and community-based organizations must collaborate to promote certification. By identifying the gaps and requirements in clinical research conducted in Portuguese centres, this study serves as a foundation for discussions on standardizing the competencies (e.g., knowledge, skills, and attitudes/behaviour) required for each professional role in clinical research (Kiilakoski and Basarab, 2022), as well as on implementing certification at the national level.

At the institutional level, a performance evaluation system, either mandatory or optional, could be introduced to evaluate the effectiveness of clinical research centres in conducting clinical studies. This could serve as an incentive to encourage improvement and adherence to best practices in clinical research development, promoting professional development and creating a more versatile and skilled workforce. Additionally, workforce structuring initiatives, such as Duke University’s effort to streamline job classifications (Brouwer et al., 2017), demonstrate the benefits of refining role definitions and career paths in clinical research. Adopting similar approaches in Portuguese clinical research centres could help optimize workforce organization, clarify career progression, and enhance competency development.

At the societal level, patient education and public awareness are crucial for accelerating the development of measures aimed at improving recruitment, adherence, and knowledge in clinical studies.

Although the survey aimed to evaluate competencies in the development and conduct of clinical studies, it shed light on the lack of experience in certain activities/responsibilities that are not conducted in the Portuguese clinical research centres. This reflects the clinical research context in Portugal, where most clinical trials are industry-driven. Hence, it is essential to encourage investigators and increase funding to promote IITs, while also enhancing the development of these competencies (Madeira et al., 2016). Thus, as a limitation of this study, using the JTF Core Competency Framework to assess competencies may not entirely align with the reality of clinical research conducted in Portugal.

Yet, this study is a pioneer in Portugal and one of the few in Europe to conduct a national survey to assess competency in conducting clinical studies, registering an impressive participation rate. However, some regions of the country conduct very few clinical trials (i.e., Inland, South, and Islands) and were not included in this assessment. This may impact the geographic representation of the sample but highlights the unequal geographic distribution of clinical studies in Portugal. On the other hand, given that the participating centres are national reference centres accounting for a high volume of clinical studies, this study serves as a good indicator of clinical research knowledge and competency in Portugal. Future studies should aim to investigate potential differences in training needs and research capacity across the entire country, including underrepresented areas, to ensure a more comprehensive national assessment.

In line with AICIB´s mission to promote, coordinate, and support activities in clinical and translational research, as well as biomedical innovation, the agency is dedicated to optimizing Portugal’s clinical, scientific, and technological potential. The findings of this study are highly valuable for AICIB, as they provide evidence to fine-tune the development of ongoing capacitation initiatives that address priority gaps and further enhance Portugal’s growing potential in clinical research.

This competency framework can serve as reference for other Portuguese clinical research centres and Portuguese speaking countries. Importantly, the proposed measures can also guide national and international centres with similar gaps. It is essential that all stakeholders, namely, industry, regulatory authorities, national agencies, clinical research centres, universities, and patient organizations, are involved in developing cohesive strategies aimed at enhancing national competencies for conducting clinical studies. This collaboration will ensure effective monitoring, secure funding, and promote long-term sustainability. By aligning their efforts in promoting training, resource allocation, regulatory and ethical surveillance, and policy implementation, these entities can not only elevate the quality of studies but also improve compliance with international standards, ultimately benefiting both researchers and patients.

## Data Availability

The raw data supporting the conclusions of this article will be made available by the authors, without undue reservation.
